# Clinical Experiences in Private Practice

**Published:** 1872-05

**Authors:** Z. C. McElroy

**Affiliations:** Zanesville Ohio


BUFFALO

### Medical and Surgical Journal.

VOL XI. MAY, 1872. No. 10.

### Original Communications.

———:o:———

ART. I.—*Clinical experiences in private practice. Complicated Chronic Case.* By Z. C. McELROY, M. D. Zanesville Ohio.

Read to Muskingum County Medical Society. April session, 1872.

Mrs. M. T., age not certainly known, but not far from 55. Native of Ireland, has resided in this country about 30 years. Has had thirteen children, seven of whom are living. Her life has been one of hardship, toil, poverty, and deprivation. Has had much of the time only the bare necessaries of life, often hardly these, and but little of its ease and comforts. Her husband died eight or ten years since in the army. His bounty at enlistment purchased a small homestead, which she still owns and occupies, and she draws the annual pension paid to the widows of privates, minus the commissions of agents. She is of small stature, and never otherwise than spare in flesh. Several of her children came to their end by violence, railroad accident, drowning, &c. Her cup of sorrow, disappointment, bereavement, poverty and want, has been through life more than filled. Personally, I have known her for many years, and have frequently rendered professional services to herself and family before, but not during the last three years. Prior to that time I had used the hypodermic syringe several times on her to relief paroxyoms of difficult breathing. [Asthma]. But while she obtained prompt relief each time it had been used, she did not like, she said, to have a *prod* used on her.

28th Dec., 1871. Has been sick for nine weeks past, and up to four or five days since has had the services of a physician all the time. He dismissed himself on account of absence from the city. She is propped up in an arm chair, and is much emaciated; breathing labored, quick and shallow; lips purplish; coughs a good deal during the day, and is able to get but little sleep for coughing at night; is not able to lie down at all some nights; passes but little urine, and what she does is very dark colored; bowels only move as she takes pills, eats very little, and drinks still less; does not drink half pint of fluid in 24 hours; has not improved any during the whole nine weeks of her present sickness; pulse good considering her condition, 90 per minute, fair volume, and regular, temperature 101. She said her previous medical attendant did not give her much encouragement as to her getting well; said that she had so many diseases that he could only treat a few of them at a time. She exhibited to me several medicines she was taking for the cure of about half her diseases. I felt curious enough to inquire of her what diseases she now had. She very naively commenced telling me their names, but as she progressed I found them so numerous that I took out my memorandum case and asked her to commence again and name them, so that I could write them down. She smiled rather incredulously, said the Doctor told her she had them, but I assured her that I was seeking information, and was afraid of my memory. I wrote them down as she named them, and they are as follows:

The Asthma, the Rheumatism, the Neuralgia, the Fevers, the Piles, the Dyspepsia, the Water brash, the Liver Complaint, her Kidneys affected, her Lungs affected, her Head affected, her Bowels affected, and threatened with the Dropsy.

Her medicines were for her Kidneys, the Piles, the Asthma, the Neuralgia, the Rheumatism, and her bowel affection.

When the list was complete I read it over to her, to be sure that all was right. She seemed a little surprised at their counting up thirteen, and did not seem to realize that she was half so bad as the list seemed to make her. I told her it appeared to me there must be some mistake about the matter, and asked her if any part of her was well. After some hesitation she said she did not think there was. "Well, then," I said "it must be that it is you that is sick, and not parts of you as the names of these diseases which I have written down indicate." "Well, I believe you are right, Doctor, it is me that is sick, and I am sick all over."

When I was a boy at school, studying arithmetic, if I worked out a sum whose figures covered my slate, and I did not get the right answer, I rubbed it all out and took a fresh start, and I studied arithmetic before the modern method of concellation was in use. Everything had to be worked out the long way. And that was the way the sum of this woman's tribulations had been worked out, and a correct answer had not been reached. So, school boy like, I said to myself, I will rub it all out and take a fresh start, with modern methods of concellation to aid me in reducing its apparent complexity to order, and abbreviate the processes of reaching the law of order which certainly underlies the apparent confusion presented in this case.

That this woman has thirteen different diseases at one and the same time, is to my mind an absurdity. The complexity it presents is all of human creation, and not according to nature, or any natural law at all. The idea of separate and distinct diseases, or indeed of diseases at all, is a human invention entirely, had its origin far back in the non-scientific past, and ought long since to have been dropped out of consideration in the study of human pathology.

In the reduction of the phenomena presented for my study by this woman's case, the first conclusion I reached was that it was her that was sick and not parts of her, or a part of her only.

Striking from the total phenomena of her body all that is not natural and there is literally nothing left, unless her mind is excepted, for it is apparently not nearly so far wrong as almost every other process of her body, though it shares her general feebleness. The temperature indicates a waste of structure in the performance of function at a higher than natural velocity, but not so high as to put in immediate danger her minute forms of structure; while on the other hand, the reproduction of her wasting structures from new material is much below the natural velocity, leaving a wide difference between the ratios of repair and waste. And besides, the very small amount of food she takes could not by any possibility supply the material necessary for the repair of her structures at their present rate of waste. Up to this time, in all probability, no positive loss of minute forms of structure have occurred in her body at other points than those concerned in the production of the phenomena called asthma. Elsewhere, apparently, the conditions and material for repair are simply absent, rather than that any permanent changes of structure exists, except, perhaps the hæmorrhoidal vessels. And for the additional reason, that a cancellation of all the phenomena she presents leaves the single prominent fact that repair is the most defective process of her body.

What are the conditions of repair? Clearly they are:

1st. Materials.

2d. Force for carrying material up through various chemical mutations to living flesh.

3d. Sufficient water to effect the solution of materials in their careers, upwards to forms of structure, and downwards and outwards, as effete matter.

Her structures, as my own, as every other thing endowed with life, animal or vegetable, in the acts of functional decay—the descent of material from chemical condition in living tissue, to the state in which it finds exit from living bodies—stores up, in one or more of the compounds then formed, the neccessary force to carry its own, and new material up to the forms of structure from which it was derived.[1] In no other possible way can personal identity be mantained through the life of a single individual, or that of a species, or a race, through centuries. And this is one of those sublimely simple laws, which shed such a flood of light on the complex phenomena of life—viz—that organic structures in the act of functional decay, store up the force for their own reproduction from new materials.

The annuals of the vegetable world—corn, rye, wheat, etc. etc.—are models of industry and fidelity to the main purpose of all life. No superior force preventing, and the conditions for their work present, they never halt a single moment in the accomplishment of the main purpose of their own, and all life, viz—to provide for

[1] Buffalo Med. & Surg. Journal, July 1871.

their own reproduction from new material. And when this purpose is accomplished, they are dead, or die soon after; for death is the parent of all life.

Of the special conditions absent in the case now under study, the absence of new material is, perhaps, the most prominent. Then there is an absence of the necessary force to carry new material up through its necessary changes to living structure, capable of per_ forming a function, and providing for its own reproduction and multiplication.

Again, there is a condition of physical motion, as well as a derangement of physical motion, incompatible, from increased velocity, with repair from new materials. Lastly, there is not sufficient fluid—that is water—to furnish the conditions for normal repair and waste.

What is my duty, as a physician, towards this woman? And what means are at my command to supply these obvious deficiencies? My duty is clearly to supply, as far as possible, these defects —dropping out of consideration any mental conception commonly associated with the term "disease," or the "cure of disease, or diseases." Not only this, but I must also drop out of consideration, in prescribing, the entire classifications of medicines, or remedial agencies now in use, for, if these several factors are admitted in the working out of the sum, or problem, a correct solution, representing, in mental conceptions, the facts actually concerned in the case will never be reached.

The indications for supplying these defects are, to retard physical motion, so as to allow her to rest and sleep in the horizontal position. Sleep is an essential condition for the repair of all of the structures.

The means to fill these indications are as follows: to procure sleep, chloral hydrate, as the material of chloral hydrate stores up a mode of force which promptly retards the rate and mode of motion in living bodies preventing sleep. But, as it contributes nothing to the sum of force available for repair, some other agent retarding motion, and at the same time supplying force and conditions for repair must be combined with it. And that which best supplies these conditions is found in opium. She is therefore to have the following. ℞ Chloral hydrate ʒiv. Sulphate of morphia gr. ij. Menstruum ℥iv. Dose, one tea spoonful every hour until she can sleep lying in bed. When the requisite amount to procure rest is ascertained, let it be one or several tea spoonsful—that will be the dose when subsequently needed.

The next defect to be supplied is material for the construction and re-construction of her various tissues. She is, therefore, to have a good diet—bread, milk, meat, butter, fish, fowl—and in a word—good living. And as she cannot be expected to take meals as in health, she is to eat as many more times per day as she would if well, at least for the present.

I have now provided for retarding motion as near to the physiological velocity as possible, and for new material. But the force supplied by her wasting structures, stored up in her lymph, will not, without some aid be competent to carry these new materials up to the forms of her living structures. That which stores up this supplemental mode of force, and is best adapted to meet the wants of the present case, is supplied by what is known in the materia medica as "peculiar bitters." For the present case, then, an ounce of prickly ash bark, in two quarts of boiling water, to be made each evening, and the whole of the infusion drank the next day. At equal times, say morning, noon and evening, she is to add to a portion of the infusion twenty to thirty drops of Aromatic sulphuric Acid. This will supply two defects—to wit—force, and water sufficient to carry on properly the work of molecular repair from new material.

There remains but one other defect to be supplied, and that is elimination. For this purpose she is to have one or more tablespoonsful solution of saline sulphates, in a tumbler of water, either cool, or very hot as she prefers, and may be taken not more frequently than every third night at bed time, or in the forenoon ℞ Sulph Magnesia ℥iv. Sulph Potass ℥iss. Sulph Ferri ʒiij. Boiling water ℥xv. Dissolve and add Elix. Vitrol ℥iss—Filter.

As my interview occurred late in the afternoon, nothing was to be taken that day or night but the chloral mixture. I knew enough of my patient's character to feel certain that my request would be punctually complied with, and as I felt reasonably certain that my expectations would be realized, I promised to call on the following Saturday.

Dec. 30th. Patient took chloral mixture as requested, slept all night, and felt herself quite a different being from what she had been the day before. Coughed a good deal after rising, but has taken food, and infusion as requested since. She is much better this morning. Breathing quite easy, little or no pain anywhere, she thinks her kidneys have got well already, as she now passes a good deal of water, not so highly colored, and without distress. Cannot say she enjoys her food, but eats it as a matter of duty.

As everything is working so satisfactorly, she is requested to continue treatment, and I am to visit her middle of next week.

Having to visit a neighbor of hers next day, she sent one of her children to request me to come and see her. The weather was very inclement and stormy. I found her in her chair again unable to lie down, breathing very much embarrassed, and is coughing almost incessantly. I gave her a tablespoonful chloral mixture, and requested her to repeat it in an hour, and every hour until she ceased coughing, and could lie down and sleep. Continue food and infusion.

January 2nd, 1872. Patient speedily got relief after I left her on Sabbath. Has been better ever since. Begins to enjoy her food, is very cheerful and pleasant. She is to continue treatment as before, taking chloral in any quantity needful to arrest coughing and procure sleep.

January 5th, 1872. Patient rapidly improved, is dressed and going about the house. Looks better, says she feels better every way. Sleeps and eats well, and thinks she is rapidly getting well.

January 13th, 1872. Patient still better. Continue treatment.

January 22d. Found patient at the wash tub. Has quite regained her usual health.

Requested her to continue the infusion a few days longer, and to take chloral when necessary.

Did not see patient again until about the middle of March. I called to get particulars of her case, and ascertain present condition. She considers herself as well as usual; attends to all her customary duties, and ceased to take any medicine some time since, excepting chloral mixture, and that only when changes of weather bring on difficult breathing, or cough, or both.

I think it reasonably certain that had I been guided in the professional management of this case by the worn out theories of life now governing the professional mind—i, e—vital force or forces, diseases, cure of diseases, the classifications of medicines, &c. I should have had about the same success as my predecessor, and he was a regular physician of good repute. I did not have to "try the effects" of this or that "remedy." I did not treat any of her so called diseases; did not cure any of her so called diseases. My attention was given to the woman, and my purpose was to supply defects, simply—in the processes needful to carry food up to normal forms of structure. And it seems to me the results justify the wisdom of my course.

An eminent living writer said, during our late civil war, that the public—that is the human—mind is slowly educated by arguments, but quickly by events.

In medical matters, it seems to me both are better than either separately. Hence, in the report of this case I have linked them together.

But it must not be understood that all chronic sickness can be thus speedily relieved. Changes of structure lie behind many of the discomforts and departures from health in the lives of old and young. For loss or complete changes in the minute forms of structure, there are no known means of restoration at the command of physicians. They must be content to make the most and best of what is left; and, an extended experience convinces me that in the general course here indicated, subject to modifications to adapt it to each individual case, the highest results attainable by medical art are realized.

And I further conclude that one of the lessons to be learned in this way is, that every complexity in organic life, pathological, or physiological, has underlying it a few simple physical laws; and that all through the wide domain of organic life, law and order reigns supreme.

